# Functional Connectivity in the Social Perception Pathway at Birth Is Linked to Attention to Faces at Four Months

**DOI:** 10.1016/j.bpsgos.2025.100597

**Published:** 2025-08-19

**Authors:** Katarzyna Chawarska, Angelina Vernetti, Huili Sun, Michelle Hampson, Chenhao Li, Suzanne Macari, Kelly Powell, R. Todd Constable, Joseph Chang, Laura R. Ment, Dustin Scheinost

**Affiliations:** a Child Study Center, Yale University School of Medicine, New Haven, Connecticut; b Department of Radiology and Biomedical Engineering, Yale University School of Medicine, New Haven, Connecticut; c Department of Statistics and Data Science, Yale University, New Haven, Connecticut; d Department of Pediatrics, Yale University School of Medicine, New Haven, Connecticut

**Keywords:** Autism, Faces, Functional connectivity, Neonates, Social perception pathway, Superior temporal sulcus

## Abstract

**Background:**

The right-lateralized social perception pathway, including the superior temporal sulcus, supports processing of dynamic, multimodal facial cues, while the right-lateralized ventral pathway, including the fusiform gyrus, is involved in processing static facial features. However, little is known about the early development of these pathways or their links to later social outcomes. In this study, we examined intrinsic functional connectivity (iFC) in these pathways in neonates with and without familial history of autism. We also investigated whether neonatal iFC was associated with reduced attention to faces at 4 months, an early autism biomarker.

**Methods:**

iFC was measured in 310 full-term, typically developing neonates from the dHCP (Developing Human Connectome Project) at 41 weeks postmenstrual age (PMA) (SD = 1.7) and in 73 full-term Yale neonates with and without a family history of autism at 44 weeks PMA (SD = 1.3). Attention to faces was assessed at 4.1 months (SD = 0.3) via eye tracking in 37 Yale participants.

**Results:**

All 4 pathways showed significant iFC (*p* < .001), with no sex differences (*p* > .159). Connectivity in the social pathway increased with age (*p* < .001). In Yale neonates, only iFC in the right social pathway was positively associated with attention to faces at 4 months (*r*_37_ = 0.456, *p* = .006). Greater attention to faces predicted fewer social concerns at 18 months (*r*_33_ = −0.358, *p* = .010).

**Conclusions:**

The right-lateralized social perception pathway represents an area of interest for identifying early neural markers of social vulnerabilities associated with autism.

## Plain Language Summary

In this study, the investigators looked at how different parts of the brain that help with social interaction and recognizing faces are connected in newborns with and without a family history of autism. The researchers studied brain activity in 383 full-term babies and later checked how much a smaller group of them paid attention to faces at 4 months of age. They found that all the brain pathways that they looked at were active, suggesting that babies are ready to process social information shortly after birth. Most importantly, stronger connections in the social brain pathway on the right side of the brain were linked to babies paying more attention to faces at 4 months, and those babies showed fewer social difficulties at 18 months. This suggests that early brain activity may help identify children who may be more likely to experience social attention vulnerabilities related to autism.

During the first 3 months of life, infants spend nearly a quarter of their waking hours viewing dynamic multimodal faces (1). Neonates prefer to look at face-like stimuli compared with carefully controlled alternatives (2) and orient more readily to speech compared with nonspeech sounds (3,4). They can also form intermodal pairings of faces and voices and recognize familiar faces only when exposed to faces that speak during the familiarization (5,6). Notably, newborns prefer faces with a direct gaze to faces with an averted gaze (7,8). They also integrate gaze and speech information and more readily recognize faces that speak and look at them directly during familiarization compared with faces that speak while looking away (9). These elementary social perception skills in neonates lay the foundation for the future development of face processing and social cognition in infancy.

**Figure 1:**
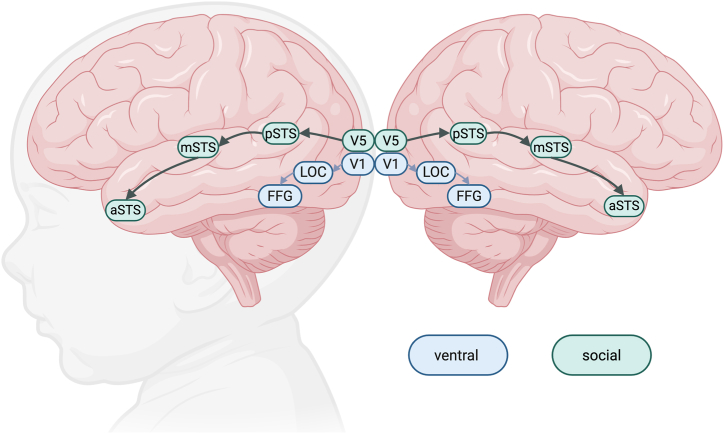
Schematic representation of the social perception and ventral face perception pathways in the brain. The social perception pathway extends from the motion-sensitive medial temporal/visual 5 (V5) area through the posterior superior temporal sulcus (pSTS), medial STS (mSTS), and anterior STS (aSTS). The ventral object/face processing pathway extends from the primary visual cortex (V1) through the lateral occipital complex (LOC) to the fusiform gyrus (FFG) area.

The processing of dynamic social information is supported by a social perception pathway that extends from the visual areas through motion-sensitive medial temporal (MT/V5) and superior temporal sulcus (STS) areas (10) (Figure 1). While face and speech-specific responses are already seen at the MT/V5 level (11), the STS specializes in dynamic aspects of social perception, including biological motion, dynamic faces, speech, and their multimodal combinations (11,12). The right-lateralized posterior STS (pSTS) is involved in face processing (11,13) and biological motion (11,14), the medial STS (mSTS) responds to speech stimuli and faces (11), and the anterior STS (aSTS) has been implicated in gaze direction perception (15) and speech (11). The STS is characterized by a posterior to anterior gradient, with more basic perceptual information represented in the posterior parts of the pathway and more abstract or conceptual representation of social stimuli moving toward the aSTS (10,16).

The STS has been an area of interest in autism for several decades. Children with autism display atypical developmental trajectories of resting-state connectivity in the STS (17). Alterations in resting-state functional connectivity (FC) between the STS and other brain areas that specialize in social perception and cognition, including the frontoparietal areas (18), inferior parietal lobule, premotor areas (18), the amygdala, ventromedial prefrontal cortex, and fusiform gyrus (FFG) (18, 19, 20, 21, 22), have been reported in older children and adults with autism. Moreover, individuals with autism display hypoactivation in the voice-selective regions of the right and left STS (23). To the best of our knowledge, only one study has examined resting-state connectivity within the social pathway in autism. Using the ABIDE (Autism Brain Imaging Data Exchange) sample, Li *et al.* (24) reported that children with autism demonstrated decreased connectivity between the right pSTS and mSTS compared with typically developing control children. The association between lower pSTS-mSTS connectivity and higher levels of autistic traits in the social domain highlights the clinical significance of this finding (24).

Due to the methodological constraints related to studying task-based brain activation during the first postnatal weeks, the neural mechanisms that underlie the early processing of social stimuli are poorly understood. It has been hypothesized that the early perceptual biases for face- versus nonface patterns are mediated by subcortical structures, including the superior colliculus and pulvinar nucleus, which are sensitive to salient features of faces, including high contrast and upper versus lower asymmetry (25,26). However, it is unlikely that the range of skills exhibited by neonates can be accounted for entirely by subcortical mechanisms (2). Task-based functional brain imaging studies utilizing functional magnetic resonance imaging (MRI) and functional near-infrared spectroscopy (fNIRS) techniques in infants suggest that 2- to 9-month-old infants activate face-selective areas, including the FFG and STS, in response to dynamic faces (27, 28, 29, 30) and biological motion (29,31). However, it remains unknown to what extent the cortical areas involved in processing multimodal social stimuli (faces, voices, biological motion) are functionally connected in neonates and associated with later face-processing skills.

We contrast the right social pathway with the contralateral pathway involving the left STS, which is concerned with highly social but largely auditory information involving speech and multimodal integration of speech and semantic concepts (32, 33, 34, 35) and gaze cues (36). This contrast is essential, as it is unclear to what extent the functional differentiation between the left and right STS is present in neonates. We also contrast the social pathway with another pathway concerned with face processing. This ventral pathway extends from the primary visual cortex (V1) through the lateral occipital complex (LOC) into the FFG (37,38) (Figure 1). The LOC contains both face- and object-selective areas, and the FFG is involved in processing static faces, including their identity and expression (39, 40, 41). Although the ventral pathway is bilateral (42), face processing in the ventral pathway is largely right lateralized, like the social pathway (43,44). Importantly, positive coactivation between V1 and FFG nodes has been reported by 27 days of age, which suggests that this is also an early maturing pathway (45). As with the STS, the FFG has been implicated in autism (46,47).

Considering the potential relevance of the social pathway for processing multimodal social cues observed in newborns (5, 6, 7,9,48), we examined for the first time its intrinsic FC (iFC) in typically developing neonates drawn from the dHCP (Developing Human Connectome Project). We hypothesized that the nodes within the right-lateralized social pathway would be positively intercorrelated at birth and that the connectivity would increase with age as a function of exposure to a social environment input (1). Subsequently, leveraging a prospective sample of Yale neonates, we examined whether FC in the social pathway was associated with selective attention to multimodal faces at 4 months. The Yale sample was enriched with participants with a familial history of autism, which is known to increase the likelihood of social vulnerabilities (49, 50, 51). Selective attention to multimodal faces represents one of the best-replicated biomarkers in autism (52,53) and is diminished in autism (54, 55, 56, 57). We hypothesized that if the strength of connectivity within the right social pathway at birth facilitates the development of visual social perception, there would be a significant association with later attention to faces in the neurodiverse sample and that this effect would not be observed in the left social pathway, which is particularly concerned with language processing, or the ventral pathway, specialized in static face processing.

## Methods and Materials

### Participants

#### dHCP Neonatal Sample

The dHCP is an observational, cross-sectional Open Science program approved by the UK National Research Ethics Authority. The dHCP participants were recruited from postnatal wards. The dHCP sample exclusion criteria were 1) a history of severe compromise at birth requiring prolonged resuscitation, 2) a diagnosed chromosomal abnormality, or 3) any contraindication to MRI scanning. In the current study, we included 310 full-term postmenstrual age (PMA) (PMA > 37 weeks) singleton neonates (49% male) with a PMA at scan from 37.4 to 44.7 weeks and an average postnatal age at scan from 0 to 5.30 weeks (Table 1). Given our focus on establishing normative neonatal patterns for the networks involved in the processing of social stimuli, the sample included only participants who completed the 18-month follow-up visit (mean age = 19.02 months, SD = 2.06, minimum [min] = 17.0, maximum [max] = 34.0) and had Bayley Scales of Infant Development scores in the cognitive and language domains >70. Similarly, due to increased familial risk factors for neurodevelopmental conditions, participants with siblings with autism or attention-deficit/hyperactivity disorder were excluded from the analysis. The exclusion was due to the structure of the dHCP data, in which these 2 risk factors were coded jointly.

**Table 1:** Sample Characteristics

	dHCP MRI Scan, *n* = 310	Yale: Neonates MRI Scan, *n* = 73	Yale: 4-Month Eye Tracking, *n* = 37
Female	159 (51%)	33 (45%)	19 (51%)
Male	151 (49%)	40 (55%)	18 (49%)
PMA at Birth, Weeks	40.13 (1.18)	39.74 (0.86)	39.86 (0.96)
PMA at Scan, Weeks	41.45 (1.65)	43.92 (1.32)	44.10 (1.20)
Age at Scan, Weeks	1.33 (1.27)	4.17 (1.24)	4.24 (1.20)
Birth Weight, kg	3.44 (0.46)	3.56 (0.39)	3.60 (0.40)
Frame-to-Frame Displacement	–	0.03 (0.02)	0.03 (0.02)
Minutes of Rest	–	11.44 (1.99)	11.36 (2.24)
Family History of Autism	0%	16 (22%)	9 (24%)
Developmental Outcome Age, Months	19.0 (2.0)	18.9 (4.0)	19.42 (3.7)
BSID Cognitive Composite	103 (9.27)	–	–
BSID Language Composite	102 (13)	–	–
Q-CHAT	27.59 (6.28)	–	–
MSEL Verbal DQ	–	97 (21)	96 (22)
MSEL Nonverbal DQ	–	103 (14)	105 (14)
ADOS-2 Total Raw Score	–	4.50 (3.42)	4.48 (3.3)
FYI Social Communication Score	–	4.20 (5.46)	5.07 (5.70)
FYI Sensory Regulatory Score	–	5.56 (6.24)	4.79 (5.49)
Diagnostic Classification			
Typical	–	43 (59%)	23 (62%)
Atypical^a^	–	19 (26%)	7 (19%)
Unknown^b^	–	11 (15%)	7 (19%)

Values are presented as *n* (%) or mean (SD).

ADOS-2, Autism Diagnostic Observation Schedule, Second Edition; BSID, Bayley Scales of Infant Development; dHCP, Developing Human Connectome Project; DQ, Developmental Quotient; FYI, First-Year Inventory; MRI, magnetic resonance imaging; MSEL, Mullen Scales of Early Learning; PMA, postmenstrual age; Q-CHAT, Quantitative Checklist for Autism in Toddlers.

a The atypical outcome group comprises of children with developmental delays including 3 children wtih autism in the full sample and 2 children with autism in the follow-up sample.

b Lost to follow-up.

#### Yale Neonatal Sample

Seventy-seven full-term (PMA > 37 weeks) singletons were scanned between April 2018 and July 2022 as part of their participation in a study of social development in infants with a familial history of autism. Four infants awoke before adequate functional data could be collected; thus, usable data were available for 73 (55% males) neonates (Table 1). Exclusion criteria were 1) congenital infections, 2) nonfebrile seizure disorder, 3) hearing loss, 4) visual impairment, 5) the presence of a known chromosomal abnormality, 6) prenatal exposure to illicit drugs, 7) a major psychotic disorder in a first-degree relative, and 8) contraindications to MRI including nonremovable metal medical implants. PMA at birth ranged from 37.6 to 41.9 weeks, the scans were conducted from 40.7 to 46.4 weeks PMA, and postnatal age at scan ranged from 1.43 to 7.00 weeks. Compared with the dHCP sample, the Yale neonates had a significantly lower PMA at birth (*p* = .009), higher PMA at scan (*p* < .001), and higher postnatal age at scan (*p* < .001). Of 73 neonates, 20 (27%) had a family history of autism in first-, second-, or third-degree relatives, which is a known risk factor for atypical social developmental outcomes, including autism or broader autism phenotype features (49, 50, 51). At follow-up, (mean = 23.63 months, SD = 2.63, min = 16.9, max = 28.0), 27% (20/73) were identified as having developmental concerns (autism: *n* = 3, specific or global developmental delays: *n* = 17), 57% (42/73) were found to be developing typically, and 15% (11/73) dropped out of the study before diagnostic assessment could take place. For comparability with the dHCP sample, behavioral outcome scores are reported based on the 18-month assessment (Table 1).

### Image Processing

For details on image registration and imaging acquisition, see the Supplement.

#### Connectivity Processing

The dHCP data were preprocessed with the dHCP functional pipeline (58), including echo distortion correction, motion correction, and independent component analysis denoising and registered to individual T2-weighted native space. The Yale data were processed using a previously validated pipeline (59). Functional images were slice-time and motion corrected using SPM8. Next, images were iteratively smoothed until the smoothness of any image had a full width at half maximum of approximately 6 mm using AFNI’s 3dBlurToFWHM. This iterative smoothing reduces motion-related confounds (60). For both datasets, all further analyses were performed using BioImage Suite (61) unless otherwise specified. Several covariates of no interest were regressed from the data, including linear and quadratic drifts (i.e., high-pass filtering with an approximate cutoff frequency = 0.003 Hz), mean cerebrospinal fluid signal, mean white matter signal, and mean gray matter signal. For additional control of possible motion-related confounds, a 24-parameter motion model (including 6 rigid-body motion parameters, 6 temporal derivatives, and these terms squared) was regressed from the data. The data were temporally smoothed with a Gaussian filter (approximate cutoff frequency = 0.12 Hz). A canonical gray matter mask defined in common space was applied to the data, so only voxels in the gray matter were used in subsequent calculations.

#### Functional Connectivity

**Figure 2:**
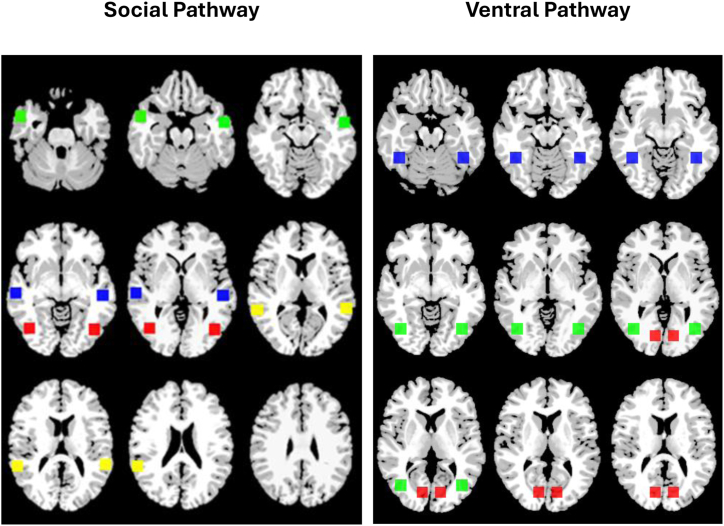
The representation of the nodes in the social (left) and ventral (right) pathways and their Montreal Neurological Institute (MNI) coordinates. The nodes in the social pathway include medial temporal/visual 5 (red), posterior superior temporal sulcus (STS) (yellow), medial STS (blue), and anterior STS (green). The nodes in the ventral pathway include the primary visual cortex (red), the lateral occipital complex (green), and the fusiform gyrus (blue). Following the radiological convention, the left side of the figures represents the right hemisphere. For MNI coordinates, see Table S1.

The coordinates of the nodes in the social and ventral networks are shown in Figure 2 and Table S1. After the seeds for each of the 3 pathways were warped from Montreal Neurological Institute space into a single participant’s space, each seed region’s time course was computed as the average time course across all voxels in the reference region. The time courses were correlated between seed pairs and transformed to *z* values using Fisher’s transformation. Following Li *et al.* (24), the intranetwork connectivity strength for each pathway was defined as the average of the individual connections within each pathway, which resulted in 4 functional connections per individual. For the social pathway, we examined connectivity between MT/V5 and pSTS, pSTS and mSTS, and mSTS and aSTS in both hemispheres to investigate how neonates process and integrate information along the pathway. The same strategy was applied for the ventral pathway in both hemispheres (V1 to LOC and LOC to FFA).

### Behaviors Associated With Autism

In the dHCP sample, behaviors associated with autism were examined at a mean age of 19.0 months (SD = 2.0) using the Quantitative Checklist for Autism in Toddlers (Q-CHAT), a 25-item screening instrument that generates a total score that captures social communication, repetitive behaviors, language, and other behaviors (62). In the Yale sample, behaviors that are associated with autism in toddlers were assessed at an average age of 18.91 months (SD = 3.38, min = 12.09, max = 26.34) using the First-Year Inventory 2.0 (FYI), a parent questionnaire that consists of Social Communication (28 items) and Sensory-Regulatory (24 items) scales (63,64) (see the Supplement for more details).

### Selective Social Attention Task

At 4.17 months (SD = 0.33, min = 3.38, max = 5.42), infants were administered the free-viewing selective social attention eye-tracking task (SSA 4.0) (56,65,66). The stimuli consisted of videos of a person speaking and looking directly at the camera and surrounded by 4 distractor toys (Figure S2); for more details, see the Supplement. The primary dependent measure was the proportion of looking at a person’s face (%Face), where the total duration of looking at the face was standardized by the total duration of looking at the scene. Of 73 neonates in the Yale sample, 37 (51%) contributed valid data to the SSA 4.0 task. Of the remaining 36 infants, 15 skipped the visit or did not participate in the SSA 4.0 procedure due to technical reasons, and 21 were excluded due to poor calibration or inattention. The retained infants did not differ from infants who were not retained in terms of PMA at scan (*p* = .217) or iFC in the right-social (*p* = .643), left-social (*p* = .084), right-ventral (*p* = .287), and left-ventral (*p* = .615) pathways at birth. They also did not differ in the verbal (*p* = .698) or nonverbal (*p* = .288) Developmental Quotient or in the FYI Social Communication (*p* = .205) or FYI Sensory Regulation (*p* = .341) scores at follow-up.

### Statistical Analysis

To examine connectivity strength within each pathway, we performed a series of *t* tests to compare the iFC values to 0. Paired *t* tests were used to compare iFC in the 6 pathways in each sample. Sex effects on iFC were examined using general linear model (GLM) analyses while controlling for PMA at scan. Exploratory analyses examining links between iFC and behavioral outcomes were performed using Pearson’s *r* correlation analysis, controlling for PMA at scan. Links between the neonatal connectome and attention were examined using Pearson’s *r* correlation coefficients and GLM while controlling for PMA at scan and frame-to-frame displacement. Raw *p* values are reported, but only the effects that survived Bonferroni correction for multiple comparisons are interpreted.

## Results

### dHCP Neonatal Sample

#### Intrinsic FC

The descriptive statistics for social and ventral pathways in the dHCP sample are presented in Table 2. The average iFC values were significantly >0 in the right-social (*t*_1,309_ = 31.92, *p* < .001), left-social (*t*_1,309_ = 27.80, *p* < .001), left-ventral (*t*_1,309_ = 19.20, *p* < .001), and right-ventral (*t*_1,309_ = 16.76, *p* < .001) pathways. All the effects remained statistically significant after Bonferroni correction for multiple comparisons (*p* < .013).

**Table 2:** Functional Within-Network Connectivity in the Right and Left Social Perception and Ventral Pathways in the dHCP and Yale Samples

Variable	dHCP Sample				Yale Sample			
	*n*	Mean (SD)	*t*	*p* Value	*n*	Mean (SD)	*t*	*p* Value
R-Social	310	0.31 (0.17)	31.92_309_	<.001	73	0.25 (0.10)	20.68_72_	<.001
L-Social	310	0.28 (0.18)	27.80_309_	<.001	73	0.39 (0.10)	34.35_72_	<.001
R-Ventral	310	0.19 (0.18)	19.20_309_	<.001	73	0.16 (0.12)	11.74_72_	<.001
L-Ventral	310	0.14 (0.15)	16.76_309_	<.001	73	0.20 (0.11)	14.75_72_	<.001

The *t* values and uncorrected *p* values represent test results comparing the pathway values to 0.

dHCP, Developing Human Connectome Project; L, left; R, right.

**Figure 3:**
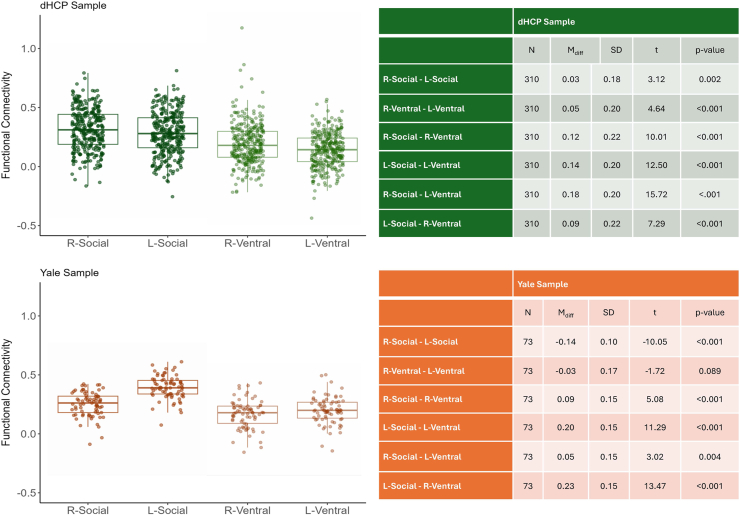
Boxplots (left) representing resting-state functional connectivity in the social and ventral pathways in the dHCP (Developing Human Connectome Project) (top) and Yale (bottom) samples. Tables (right) reporting pairwise comparisons between the edges in the social and ventral pathways in the dHCP (top) and Yale (bottom) samples. L, left; R, right.

Pairwise comparisons between the pathways are reported in Figure 3. Both left and right social pathways showed significantly higher connectivity than the ipsilateral and contralateral left and right ventral pathways (all *p* values < .001). Also, the right-lateralized social and ventral pathways had higher connectivity than the corresponding left-lateralized pathways (all *p* values < .002). All the effects remained statistically significant after Bonferroni correction for multiple comparisons (*p* < .017).

#### Sex Differences

A series of GLM analyses indicated that there were no significant effects of sex for any of the pathways (all *p* values > .149) (Table 3).

**Table 3:** Comparisons of Intrinsic Functional Connectivity Values in the Social and Ventral Pathways in Male and Female Neonates in the dHCP and Yale Samples While Controlling for Postmenstrual Age at Scan

Pathway	Female		Male		*F*	*p* Value
	*n*	Mean (SD)	*n*	Mean (SD)		
dHCP Sample						
R-social	159	0.32 (0.17)	151	0.31 (0.17)	0.01	.929
L-social		0.27 (0.17)		0.29 (0.19)	2.00	.159
R-ventral		0.20 (0.17)		0.19 (0.19)	0.81	.811
L-ventral		0.15 (0.14)		0.13 (0.15)	0.44	.277
Yale Sample						
R-social	33	0.26 (0.11)	40	0.24 (0.10)	0.48	.493
L-social		0.39 (0.10)		0.39 (0.10)	0.00	.956
R-ventral		0.16 (0.12)		0.17 (0.12)	0.02	.369
L-ventral		0.20 (0.08)		0.20 (0.14)	0.03	.849

dHCP, Developing Human Connectome Project; L, left; R, right.

#### Associations With PMA at Scan and Outcomes

iFC in the right-social (*r*_310_ = 0.21, *p* < .001) and left-social (*r*_310_ = 0.15, *p* = .007) pathways showed statistically significant correlations with PMA at scan (Table 4). There was also a significant correlation between the right-ventral pathway and PMA at scan (*r*_310_ = 0.12, *p* = .030) but not the left-ventral pathway (*r*_310_ = 0.05, *p* = .369). Only the correlations observed in the left and right social pathways remained significant after Bonferroni correction for multiple comparisons (*p* < .017). Lastly, in an exploratory manner, we examined associations between brain connectivity and the Q-CHAT scores at outcome while controlling for PMA at scan. There was a significant correlation between total Q-CHAT scores and the right-social (*r*_306_ = −0.133, *p* = .020) and left-social (*r*_306_ = −0.114, *p* = .046) pathways but not with the right-ventral (*r*_306_ = −0.039, *p* = .499) or left-ventral (*r*_306_ = −0.056, *p* = .332) pathways. While they are suggestive, none of the correlations survived Bonferroni correction for multiple comparisons (*p* < .013).

**Table 4:** Pearson’s *r* Correlation Coefficients Examining Associations Between PMA at the Time of the Scan and Intrinsic Functional Connectivity in the Social and Ventral Pathways in the dHCP and Yale Samples

Pathway	Statistic	PMA at Scan	
		dHCP, *n* = 310	Yale, *n* = 73
R-Social	*r*	0.21	0.203
	*p*	<.001^a^	.085
L-Social	*r*	0.15	−0.017
	*p*	.007^a^	.885
R-Ventral	*r*	0.12	0.017
	*p*	.030	.890
L-Ventral	*r*	0.05	0.036
	*p*	.369	.765

dHCP, Developing Human Connectome Project; L, left; PMA, postmenstrual age; R, right.

a Effects that survived Bonferroni correction for multiple comparisons.

### Yale Neonatal Sample

#### iFC, Sex Effects, Associations With Age and Social Outcomes

Similarly, as in the dHCP sample, in the Yale sample, there was robust evidence of iFC between the nodes of the right-social (*t*_72_ = 20.68, *p* < .001), left-social (*t*_72_ = 34.35, *p* < .001), right-ventral (*t*_72_ = 11.74, *p* < .001), and left-ventral (*t*_72_ = 14.75, *p* < .001) pathways (Table 2). Also, as in the dHCP sample, there were no significant effects of sex on iFC in any of the pathways (Table 3). Lastly, associations with PMA at scan were not statistically significant in the smaller Yale sample. However, the positive association between the right-social pathway and PMA showed a magnitude similar to the effect in the dHCP sample and a trend toward statistical significance (Table 4). As in the dHCP sample, we examined a direct correlation between the connectivity measures at birth and 18-month social communication outcomes. In this smaller sample, the associations were not statistically significant (all *p* values > .312); however, the magnitude of the effect for the right-social pathway (*r*_67_ = −0.126, *p* = .312) was similar to that observed in the dHCP sample.

Pairwise comparisons between the pathways are reported in Figure 3. Similarly, as in the dHCP sample, both the left and right social pathways showed significantly higher connectivity than the ipsilateral and contralateral left and right ventral pathways (all *p* values < .004). However, the right-social pathway had lower connectivity than the left-social pathway (*p* < .001), and the right-ventral pathway was not significantly different from the left-ventral pathway (*p* = .089). All the effects remained statistically significant after Bonferroni correction for multiple comparisons (*p* < .017).

#### Associations Between the iFC Pathways and Social Attention at 4 Months

**Figure 4:**
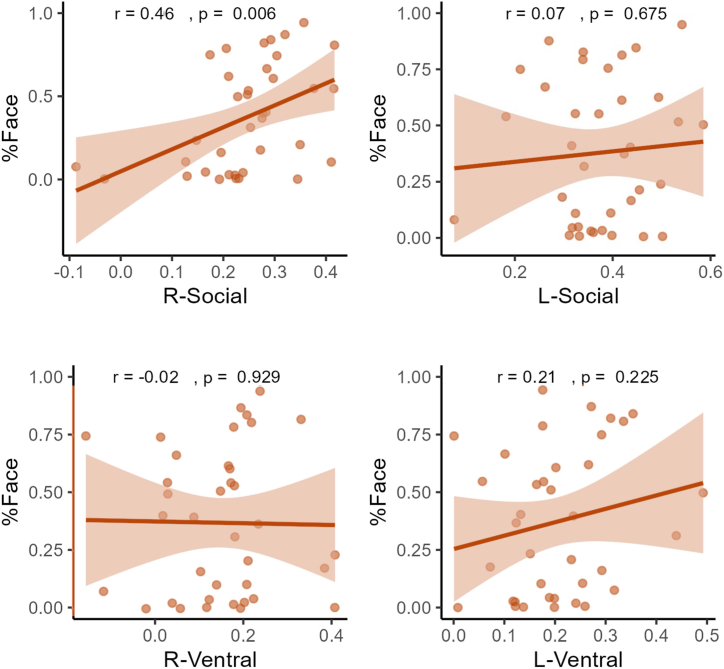
Pearson’s *r* correlation coefficient plots representing associations between proportion of looking at the face at 4 months (%Face) and functional connectivity in the social and ventral pathways in the Yale sample. L, left; R, right.

At 4 months, the infants spent 67% (SD = 24) of the time attending to the visual scene and 37% (SD = 37) looking at the face on average. After controlling for PMA at scan and frame-to-frame displacement, greater iFC in the right-social pathway was positively associated with attention to dynamic faces at 4 months (*r*_37_ = 0.456, *p* = .006) (Figure 4), and the effect remained significant after Bonferroni correction for multiple comparisons (*p* < .013). Neither PMA at scan nor frame-to-frame displacement contributed significantly to the models (see Table S3). There was a significant association between %Face at 4 months and the FYI Social Communication score at 18 months (*r*_33_ = −0.358, *p* = .041) but not with the FYI Sensory Regulation score (*r*_31_ = −0.058, *p* = .746). There were no significant associations between the left-social (*r*_37_ = 0.074, *p* = .675), right-ventral (*r*_7_ = −0.015, *p* = .929), or left-ventral (*r*_37_ = 0.210, *p* = .225) pathways and social attention at 4 months.

## Discussion

Newborns evidence relatively advanced skills in processing multimodal faces (2,5, 6, 7,48,67). Here, we demonstrated that the cortical areas located along the right social pathway, which specialize in processing multimodal faces (10), showed strong within-network connectivity shortly after birth. This connectivity did not vary by sex but increased with age, presumably due to exposure to the newborns’ rich social environment. Positive within-pathway connectivity was also found for the bilateral ventral pathways involved in face recognition. The presence of such robust intercorrelations at birth suggests presence of an experience-expectant functional infrastructure that enables newborns to select and integrate sensory information in service of learning and adaptation to their social environment. These results extend previous fNIRS (68) and electroencephalography (69) studies that found that broadly defined temporal cortices activate preferentially in response to faces shortly after birth. Moreover, we demonstrated, for the first time, that variability in FC within the right-lateralized social pathway predicted individual differences in social attention in early infancy. This effect was absent in the control pathways, including the right-lateralized ventral pathway. Thus, as in older individuals (38,70), the social and ventral pathways appear to be functionally distinct in neonates. The study offers the first evidence of a social perception pathway highly relevant for survival in neonates and its significance for the development of social attention.

The current findings shed light on the neural bases of social perception in typically developing newborns and contribute to understanding the brain processes underlying one of the best-replicated biomarkers in autism: limited attention to multimodal faces. It has been reported in presymptomatic infants later diagnosed with autism (54, 55, 56), in toddlers (57,65,66), and in school-age children with autism (52,53). We demonstrated for the first time that attention to multimodal faces at 4 months relates back to the strength of connectivity within the right social perception pathway at birth. The first few months of life are crucial for the emergence of foundational social cognition and communication skills (71, 72, 73). Here, we demonstrated that individual differences in social attention at 4 months predicted later social functioning at 18 months. We hypothesize that better connectivity within the social pathway facilitates attention to and processing of complex multimodal faces in the months following birth and lays the foundation for more sophisticated social cognitive skills emerging during the second year of life. The significance of our findings to autism is further strengthened by a recent report that underconnectivity between the nodes in the right social perception pathway in school-age children tracks with social autistic traits across the spectrum of neurodevelopment (24).

This study adds to the growing evidence linking the characteristics of functional brain connectivity at birth to genetic risk factors for autism (74, 75, 76) and later behavioral expression of autistic traits (76). Specifically, it has been reported that hypoconnectivity between the left-lateralized anterior insula and the amygdala tracks with greater social communication concerns related to autism at 12 to 18 months in children with genetic risk factors for autism (76). Given the role that the 2 brain regions play in human bonding (77), alterations in communication between them may contribute to weakening the very mechanism that supports the development of social engagement and motivation. Here, we demonstrated that lower FC in the right-lateralized social pathway involving the STS at birth related to a less robust ability to select dynamic multimodal faces for processing 4 months later, a vulnerability that may undermine participation in and learning from face-to-face interactions. Considering that functional brain connectivity is an intermediary between genes and behavior (78), altered network characteristics at birth are essential for determining processes that enhance risk for future onset of behavioral symptoms (79). Findings that individual differences in neonatal brain connectivity associated with later behaviors relevant to autism should encourage future studies of links between early brain development and psychopathology.

Compared with the links to proximal outcomes (eye tracking), links between brain connectivity and distal outcomes (autism screeners) were modest. To the best of our knowledge, only one imaging study using fNIRS has examined links between the recruitment of the superior temporal cortices in response to faces in 7-month-old typically developing infants and sociability at 18 months and has also reported modest associations (30). It is likely that the properties of the neonatal connectome support more proximal social accomplishments, which in turn facilitate the development of more sophisticated social cognitive skills at 18 months. Moreover, the field would benefit from studies that examine the links between the neonatal connectome and longer-term outcomes that represent specific skills relevant to the network of interest in samples enriched with individuals at risk for developing specific psychopathologies.

### Limitations and Future Directions

Although the overall characterization of the social pathway in neonates is based on a large sample of typically developing neonates, the associations between the pathway and social attention were assessed in a smaller sample, highlighting the necessity for replication in a larger sample enriched with autistic participants. The dHCP and Yale samples differed in age at scan, the composition of the samples, and data acquisition and preprocessing parameters. Despite these differences, the within-group between-network comparisons were very similar in the 2 samples, except for the comparisons between the left and right social and ventral pathways. Given that the Yale neonates had significantly higher PMA and postnatal age at scan, the latter differences may be due to differential pruning rate or sensitivity to environmental input in the right and left face perception pathways. The significance of these findings remains to be clarified, particularly through longitudinal studies that span the fetal to neonatal transition and extend into infancy, when the target networks are shaped in response to environmental inputs.

### Conclusions

The current study demonstrates that a functional brain pathway tuned to multimodal social stimuli is evolutionarily optimized to ensure early survival at birth and is robustly associated with later social outcomes relevant to autism.

## Supplementary Material

Supplemental Text and Tables S1–S2

Key Resources Table
