# Supplementary material for: Functional Connectivity in the Social Perception Pathway at Birth Is Linked to Attention to Faces at Four Months
Source: Biol Psychiatry Glob Open Sci. 2025 Aug 19;5(6):100597. doi: 10.1016/j.bpsgos.2025.100597 (PMC12538059; doi:10.1016/j.bpsgos.2025.100597)
Supplement: Supplemental Text and Tables S1–S2 [file mmc1.pdf]

SUPPLEMENTARY INFORMATION

Functional Connectivity in the Social Perception Pathway at Birth Is Linked With Attention to Faces at 4 Months

Chawarska *et al.*

Supplemental Materials

Image Registration

For the dHCP data, the dHCP functional pipeline was used to align the functional images to the dHCP template space[1]. For the Yale data, functional images were aligned to a custom infant template using a series of linear and nonlinear registrations. Images are aligned to the template space using a 12 parameter affine registration by maximizing the normalized mutual information between individual scans and the template brain. These aligned images were averaged together to form the initial template for non-linear registration. Images were non-linearly registered to an evolving group average template in an iterative fashion using a previously validated algorithm (Scheinost et al, 2017). This algorithm iterates between estimating a local transformation to align individual brains to a group average template and creating a new group average template based on the previous transformations. The local transformation was modeled using a free-form deformation (FFD) parameterized by cubic B-splines. This transformation deforms an object by manipulating an underlying mesh of control points. The deformation for voxels in between control points was interpolated using B-splines to form a continuous deformation field. Positions of control points were optimized using conjugate gradient descent to maximize the normalized mutual information between the template and individual brains. After each iteration, the quality of the local transformation was improved by increasing the number of control points and decreasing the spacing between control points to capture a more precise alignment. A total of 4 iterations were performed with decreasing control point spacings of 15 mm, 10 mm, 5 mm, and 2.5 mm. To help prevent local minimums during

optimization, a multi-resolution approach was used and 3 resolution levels were used at each iteration. These registrations were calculated independently and combined into a single transform. This approach allows the participant images to be transformed into a common space with only one transformation, reducing interpolation error. Functional images were linearly registered to the anatomical image, which was nonlinearly registered to the infant template using a previously validated algorithm.[2] Similarly, the same algorithm registered the infant templates for the dHCP and Yale datasets to the MNI brain. Please see Holmes et al., for more details regarding this appraoch[3].

Imaging Acquisition

dHCP Cohort. Imaging was acquired at the Evelina Newborn Imaging Centre, Evelina London Children's Hospital, using a 3T Philips Achieva system (Philips Medical Systems). All infants were scanned without sedation in a scanner environment, including a dedicated transport system, positioning device, and a customized 32-channel receiver head coil with a custom-made acoustic hood. MRI-compatible ear putty and earmuffs were used to provide additional acoustic noise attenuation, and infants were fed, swaddled, and positioned in a vacuum jack before scanning to facilitate natural sleep.[4] High temporal resolution multiband EPI (TE=38 ms; TR=392 ms; MB factor=9x; 2.15 mm isotropic) specifically developed for neonates was acquired for 15 min.

Yale Neonatal Project. Participants were scanned without sedation during natural sleep using the feed-and-wrap protocol.[5] Infants were fed, bundled with multiple levels of ear protection, and immobilized in an MRI-safe vacuum swaddle. Heart rate and O_2 saturation were continuously monitored during all scans. The scans used a 3T Siemens

(Erlangen, Germany) Prisma MR system with a 32-channel parallel receiver head coil. Functional runs were acquired using a multiband T2*-sensitive gradient-recalled, single-shot echo-planar imaging pulse sequence (TR = 1 s, TE = 31 ms, FoV = 185 mm, flip angle 62°, multiband = 4, matrix size 92 × 92). Each volume consisted of 60 slices parallel to the bi-commissural plane (slice thickness 2 mm, no gap). We collected 4-5 functional runs, each comprising 360 volumes. The mean frame-to-frame displacement was calculated for each run for every individual, and runs with a mean frame-to-frame displacement greater than 0.10 mm were removed from further analysis. Each neonate had on average 11.4 min (SD = 2.0) of usable functional data with an average frame-to-frame displacement of 0.03 (SD=0.02, Min: 0.01, Max: 0.10). High-resolution T1-and T2-weighted 3D anatomical scans were acquired using an MPRAGE sequence (TR=2400 ms, TE=1.18 ms, flip angle=8°, thickness=1 mm, in-plane resolution=1 mm x 1 mm, matrix size=256 × 256) and a SPACE sequence (TR=3200 ms, TE=449 ms, thickness=1 mm, in-plane resolution=1 mm x 1 mm, matrix size=256 × 256).

Assessment of Behaviors Associated with Autism in the Second Year of Life.

In the dHCP sample, behaviors associated with autism were examined at mean age of 19.0 months (SD=2.0) using the Quantitative Checklist for Autism in Toddlers (Q-CHAT), a 25-item screening instrument which generates a total score capturing a broad range of behaviors relevant to autism including social communication, repetitive behaviors, language and other behaviors.[6] In the Yale sample, behaviors that are associated with autism in toddlers were assessed at an average age of 18.91 months (SD=3.38, Min=12.09,

Max=26.34) using the First Year Inventory 2.0 (FYI), a parent questionnaire consisting of the Social Communication (28 items) and Sensory-Regulatory (24 items) scales.[7,8] The FYI measure is sensitive to autism-specific behaviors in the general population and amongst infants with a family history of autism,[9-13] and shows positive correlations with concurrent measures of autism symptoms based on the ADOS-2 Toddler Module[14] or the Autism Observation Scale for Infants (AOSI).[15] Unlike the ADOS-2 and AOSI, the FYI was designed to quantify autism traits in the general population and, thus, is an appropriate measure for neurodiverse samples. Risk scores were generated based on assigned risk points derived from the normative sample.[7] Each scale scores range from 0 to 50 on a semi-logarithmic scale, with higher scores indicating more symptoms.

Social Attention Task at 4 months

At M=4.2 (SD=0.4) months, infants were administered the free-viewing selective social attention eye-tracking task, version 4.0 (SSA 4.0).[16-18] The stimuli consisted of four 8-second videos of a person speaking and looking directly at the camera and surrounded by four distractor toys (**Figure 3**. Each session began with a short cartoon video followed by a 5-point calibration procedure. Each infant was presented with 4 8-second trials of a stimulus where a person spoke using child-directed speech while looking directly at the camera. The trials were separated by 500 millisecond breaks consisting of a black screen with no fixation cross or attention-getters. Sessions were conducted in a dark, sound-proof room with a 24" widescreen LCD monitor. Infants sat in a high-chair approximately 60 centimeters from the screen. To evaluate the overall attention to the task, we computed a

proportion of time they spent watching the scene standardized by the total duration of the task (%Valid). The primary dependent measure was the proportion of looking at a person's face (%Face), where the total duration of looking at the face was standardized by the total duration of looking at the scene. To be included in the %Face analysis, the children had to attend to the scene for at least 20% of the time (%Value > 20%).

Table S1. Mean (SD) pairwise correlations between the nodes within the social perception and ventral pathways in the dHCP and Yale neonatal samples. The uncorrected p - values represent test results comparing the between - node correlation values to 0.

**dHCP sample**					
	Variable	N	Mean	SD	p - value
Social Pathways	R MT/V5 – R pSTS	310	0.07	0.19	<0.001
	R pSTS – R mSTS	310	0.53	0.29	<0.001
	R mSTS – R aSTS	310	0.34	0.25	<0.001
	L MT/V5 – L pSTS	310	0.06	0.21	<0.001
	L pSTS – L mSTS	310	0.41	0.28	<0.001
	L mSTS – L aSTS	310	0.37	0.26	<0.001
Ventral Pathways	L V1 – L LOC	310	0.05	0.19	<0.001
	L LOC – L FFG	310	0.23	0.23	<0.001
	R V1 – R LOC	310	0.11	0.24	<0.001
	R LOC – R FFG	310	0.27	0.25	<0.001
**Yale Sample**					
	Variable	N	Mean	SD	p - value
Social Pathways	R MT/V5 – R pSTS	73	0.03	0.17	0.188
	R pSTS – R mSTS	73	0.48	0.20	<0.001
	R mSTS – R aSTS	72	0.24	0.16	<0.001
	L MT/V5 – L pSTS	73	0.08	0.14	<0.001
	L pSTS – L mSTS	73	0.48	0.19	<0.001
	L mSTS – L aSTS	73	0.62	0.16	<0.001
Ventral Pathways	L V1 – L LOC	73	0.09	0.18	<0.001
	L LOC – L FFG	72	0.31	0.14	<0.001
	R V1 – R LOC	73	0.05	0.14	0.003
	R LOC – R FFG	73	0.28	0.18	<0.001

Table S2. General linear model analysis examining contribution of the functional connectivity values at birth in the R-Social, L-Social, R-Ventral and L-Ventral pathways to the proportion of looking at faces at 4 months, while controlling for the effects of PMA at scan and frame-to-frame displacement in the Yale sample.

Parameter	Estimate	Standard Error	t Value	Pr > |t|
Intercept	1.00	1.96	0.51	0.613
**R_Social**	**1.43**	**0.48**	**2.95**	**0.006**
PMA_scan	-0.02	0.04	-0.51	0.611
FtF displacement	0.74	2.42	0.31	0.761

Parameter	Estimate	Standard Error	t Value	Pr > |t|
Intercept	0.25	2.18	0.11	0.910
**L-Social**	0.23	0.53	0.42	0.675
PMA_scan	0.00	0.05	0.02	0.982
FtF displacement	-0.32	2.68	-0.12	0.906

Parameter	Estimate	Standard Error	t Value	Pr > |t|
Intercept	0.22	2.21	0.1	0.923
**R-Ventral**	-0.04	0.45	-0.09	0.929
PMA_scan	0.00	0.05	0.08	0.937
FtF displacement	-0.34	2.71	-0.12	0.902

Parameter	Estimate	Standard Error	t Value	Pr > |t|
Intercept	0.07	2.14	0.03	0.975
**L-Ventral**	0.64	0.52	1.24	0.225
PMA_scan	0.00	0.05	0.1	0.921
FtF displacement	-1.12	2.70	-0.41	0.682

1. Fitzgibbon SP, Harrison SJ, Jenkinson M, et al. The developing Human Connectome Project (dHCP) automated resting-state functional processing framework for newborn infants. *Neuroimage*. 12 2020;223:117303. doi:10.1016/j.neuroimage.2020.117303
2. Scheinost D, Kwon SH, Lacadie C, et al. Alterations in Anatomical Covariance in the Prematurely Born. *Cerebral Cortex*. 2017;27(1):534-543. doi:10.1093/cercor/bhv248
3. Holmes CJ, Hoge R, Collins L, Woods R, Toga AW, Evans AC. Enhancement of MR Images Using Registration for Signal Averaging. *Journal of Computer Assisted Tomography*. 1998;**22**(2):324-333.
4. Eyre M, Fitzgibbon SP, Ciarrusta J, et al. The Developing Human Connectome Project: typical and disrupted perinatal functional connectivity. *Brain*. Mar 17 2021;doi:10.1093/brain/awab118
5. Kwon SH, Scheinost D, Lacadie C, et al. GABA, Resting-State Connectivity and the Developing Brain. *Neonatology*. Jun 26 2014;106(2):149-155. doi:10.1159/000362433
6. Allison C, Baron-Cohen S, Wheelwright S, et al. The Q-CHAT (Quantitative CHecklist for Autism in Toddlers): A normally distributed quantitative measure of autistic traits at 18-24-months of age: Preliminary report. *Journal of Autism and Developmental Disorders*. Sep 2008;38(8):1414-1425.
7. Reznick J, Baranek GT, Reavis S, Watson LR, Crais ER. A parent-report instrument for identifying one-year-olds at risk for an eventual diagnosis of autism: The First Year Inventory. *Journal of Autism and Developmental Disorders*. 2007;37(9):pp.
8. Baranek GT, Watson LR, Crais ER, Reznick JS. *First-Year Inventory (FYI) 2.0*. 2003.
9. Stephens RL, Sabatos-DeVito M, Reznick JS. The development and validation of attention constructs from the First Year Inventory. *Psychological assessment*. 2017;29(5):568.
10. Watson LR, Baranek GT, Crais ER, Reznick J, Dykstra J, Perryman T. The First Year Inventory: Retrospective parent responses to a questionnaire designed to identify one-year-olds at risk for autism. *Journal of Autism and Developmental Disorders*. 2007;37(1):pp.
11. Rowberry J, Macari S, Chen G, et al. Screening for autism spectrum disorders in 12-month-old high-risk siblings by parental report. *Journal of autism and developmental disorders*. 2015;45(1):221-229.
12. Turner-Brown LM, Baranek GT, Reznick JS, Watson LR, Crais ER. The First Year Inventory: a longitudinal follow-up of 12-month-old to 3-year-old children. *Autism*. 2013;17(5):527-540.
13. Meera SS, Donovan K, Wolff JJ, et al. Towards a Data-Driven Approach to Screen for Autism Risk at 12 Months of Age. *Journal of the American Academy of Child & Adolescent Psychiatry*. 2021;60(8):968-977.
14. Macari SL, Wu GC, Powell KK, Fontenelle S, Macris DM, Chawarska K. Do parents and clinicians agree on ratings of autism-related behaviors at 12 months of age? A study of infants at high and low risk for ASD. *Journal of autism and developmental disorders*. 2018;48(4):1069-1080.
15. Lee HY, Vigen C, Zwaigenbaum L, et al. Construct validity of the First-Year Inventory (FYI Version 2.0) in 12-month-olds at high-risk for Autism Spectrum Disorder. *Autism*. 2021;25(1):33-43.

16. Shic F, Wang Q, Macari SL, Chawarska K. The role of limited salience of speech in selective attention to faces in toddlers with autism spectrum disorders. *Journal of Child Psychology and Psychiatry*. 2020;61(4):459-469.
17. Chawarska K, Macari S, Shic F. Decreased Spontaneous Attention to Social Scenes in 6-Month-Old Infants Later Diagnosed with Autism Spectrum Disorders. *Biological Psychiatry*. 2013;74(3):195-203.
18. Chawarska K, Macari S, Shic F. Context modulates attention to social scenes in toddlers with autism. *Journal of Child Psychology and Psychiatry*. 2012;53(8):903-913. doi:10.1111/j.1469-7610.2012.02538.x
